# Validation of a 3D CT method for measurement of linear wear of acetabular cups: A hip simulator study

**DOI:** 10.3109/17453674.2011.552777

**Published:** 2011-02-10

**Authors:** Anneli Jedenmalm, Fritjof Nilsson, Marilyn E Noz, Douglas D Green, Ulf W Gedde, Ian C Clarke, Andreas Stark, Gerald Q Maguire, Michael P Zeleznik, Henrik Olivecrona

**Affiliations:** ^1^Department of Clinical Sciences, Biomaterial- and Biomechanical Laboratory, Lund University, Lund, Sweden; ^2^Department of Fiber and Polymer Technology, School of Chemical Science and Engineering, Royal Institute of Technology, Stockholm, Sweden; ^3^Department of Radiology, New York University School of Medicine, New York, NY, USA; ^4^Orthopaedic Research Center, Loma Linda University, Loma Linda, CA, USA; ^5^Department of Molecular Medicine and Surgery, Karolinska Institute, Stockholm, Sweden; ^6^School of Information and Communication Technology, Royal Institute of Technology, Stockholm, Sweden; ^7^Saya Systems Inc., Salt Lake City, UT, USA

## Abstract

**Background:**

We evaluated the accuracy and repeatability of a 3D method for polyethylene acetabular cup wear measurements using computed tomography (CT). We propose that the method be used for clinical in vivo assessment of wear in acetabular cups.

**Material and methods:**

Ultra-high molecular weight polyethylene cups with a titanium mesh molded on the outside were subjected to wear using a hip simulator. Before and after wear, they were (1) imaged with a CT scanner using a phantom model device, (2) measured using a coordinate measurement machine (CMM), and (3) weighed. CMM was used as the reference method for measurement of femoral head penetration into the cup and for comparison with CT, and gravimetric measurements were used as a reference for both CT and CMM. Femoral head penetration and wear vector angle were studied. The head diameters were also measured with both CMM and CT. The repeatability of the method proposed was evaluated with two repeated measurements using different positions of the phantom in the CT scanner.

**Results:**

The accuracy of the 3D CT method for evaluation of linear wear was 0.51 mm and the repeatability was 0.39 mm. Repeatability for wear vector angle was 17°.

**Interpretation:**

This study of metal-meshed hip-simulated acetabular cups shows that CT has the capacity for reliable measurement of linear wear of acetabular cups at a clinically relevant level of accuracy.

Debris due to polyethylene wear may trigger aseptic loosening in total hip arthroplasty (THA) (Santavirta et al. 1995, Mohanty 1996). With the methods currently available, it is difficult to determine small amounts of wear in vivo without using invasive methods such as radiostereometric analysis (RSA) (Schewelov et al. 2004). Conventional radiography is the most common routine clinical method, but it yields only 2D results with low accuracy—of about 4 mm (Clarke et al. 1976). Several 3D reconstruction methods exist, such as AP radiographs, but these methods are still too complicated to be reliable in a routine clinical setting and the accuracy tends to be lower in a clinical situation than under laboratory conditions (Clarke et al. 1976).

Current multislice CT scanners, which offer accurate spatial volume resolution in both 2D and 3D without substantial distortion, are non-invasive and fast. Metal artifacts from the implant are suitably suppressed by software algorithms from the CT manufacturers. We have previously shown that CT can also be used for evaluation of acetabular cup position and migration (Olivecrona et al. 2002, 2003c, 2004). A retrieval study showed that CT can also be used for evaluation of 3D penetration of the femoral head into metal-backed acetabular cups with an accuracy of 1 mm (Olivecrona et al. 2005). Based on that study, we developed a new approach for wear assessment using CT and it was shown to achieve an accuracy of 0.6 mm and a repeatability of 0.4 mm (Olivecrona et al. 2005). This new approach relies on placing several landmarks in the 3D CT volume on the surfaces of the head and cup. Spheres are then fitted to these data points, and from these spheres the femoral head penetration can be calculated.

As part of this study, the method described was developed futher—mainly with improved software that allowed many more landmark points to be placed on the 3D surfaces, and the possibility of comparing the same implant at 2 time points (e.g. pre-wear and post-wear). A model study was performed with a hip simulator in order to estimate the accuracy and reproducibility of the method proposed. Both gravimetric results and measurement results from a coordinate measurement machine (CMM) were used as reference methods. We used a different cup design with a metal mesh molded into the polyethylene outer surface, in order to avoid back-side wear.

Due to an unanticipated upgrade of the CT software, we also simulated the clinical situation where a CT scan is done postoperatively and then at a follow-up several years later, by using a different CT scan protocol before and after wear.

## Material and methods

**Figure 1.**
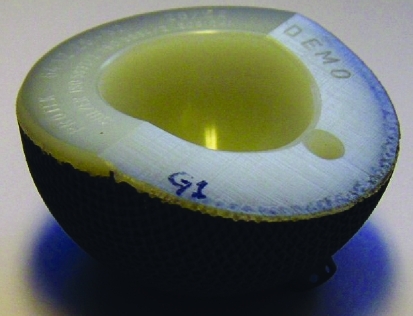
Polyethylene acetabular cup with titanium mesh molded on the outside.

We studied 12 commercial ultra-high molecular weight (GUR1020 UHMWPE) cups (Zimmer) with a molded titanium mesh on the outside (Figure 1). According to the manufacturer, the inner diameter was 28 mm, whereas the outer diameter ranged from 48 to 64 mm. The femoral heads were Durasul cobalt-chromium (CoCr) heads (Zimmer). They were manually roughened with P320-grit SiC paper, producing circular multidirectional wear tracks before the tests in order to increase the wear rate. The clearance between head and cup diameter was measured with a coordinate measurement machine (CMM) (Wenzel LH44; Wenzel, Wiesthal, Germany; accuracy in 3D: (3 + L/300) μm; maximum measured deviation at calibration: 2.4 μm) and was estimated to be 0.51 mm (SD 0.01).

All cups were measured using the CMM, a microbalance (resolution: ± 0.01 mg; accuracy: ± 0.1 mg; Sartorius AG, Göttingen, Germany) and CT scanner (LightSpeed QX/I; General Electric Medical Systems, Milwaukee, WI) before and after wear testing in a hip simulator (Shore Western, Monrovia, CA).

### Hip simulator wear testing with gravimetric measurement

All cups were presoaked in distilled water for 4 weeks before wear testing, in order to stabilize the soak rate. 8 cups were wear tested together with femoral heads in a hip simulator, with bovine serum as lubricant (a 10% solution in order to increase the wear rate (Wang et al. 1998, 2004)) until a total wear of at least 200 mg was reached. Weighing was performed after 0, 0.4, 1.15, 1.65, 2.5, and 2.8 million cycles (Mc). 4 cups (2 unsterile and 2 sterilized) were used as soak controls in serum. Wear rate was determined by weighing all cups, and it was corrected for soak. Briefly, the cups were ultrasonically cleaned, blow-dried with nitrogen gas, vacuum dried for 30 min, and then weighed 4 times in rotation with the microbalance at room temperature. Wear volume was calculated by dividing the gravimetric data by the density of the cups (935 kg/m^3^).

### Wear testing with measurement by CT examination

A model made of polymethyl methacrylate was used to fix the implant in an anatomical position at a 45° angle to the sagittal plane. A titanium stem (attached to this model) was used to fix the femoral head in position in the implant. Pen marks were then used to allow recreation of this relative orientation of the implant and femoral head at different measurement times.

2 CT examinations of each implant were done. The data volumes were acquired with 1.25 mm collimation and a pitch of 3 (i.e. 0.75 mm/rotation) at 120 kV. The current was 40 mA at the measurement before wear, and then changed to 100 mA at measurement after wear, due to an unanticipated CT software upgrade that can well happen in the clinical situation with hospital-based CT units—since it is common to change acquisition parameters and upgrade CT software on an annual basis. Slices were reconstructed at 1.25-mm increments. The implants were scanned in approximately the same positions as when implanted in a patient. To mimic different patient positions, the position of the model in the CT was altered between the scans. The changes in position of the model were larger than would be expected with patients. The height of the table was 81–83 cm, and it was not changed from scan to scan or during a scan.

### CT image analysis

The CT method developed had 2 distinct parts: (1) image analysis tools for obtaining CT coordinate data, and (2) locally developed software for determining linear wear and wear angles from the coordinate data.

**Figure 2.**
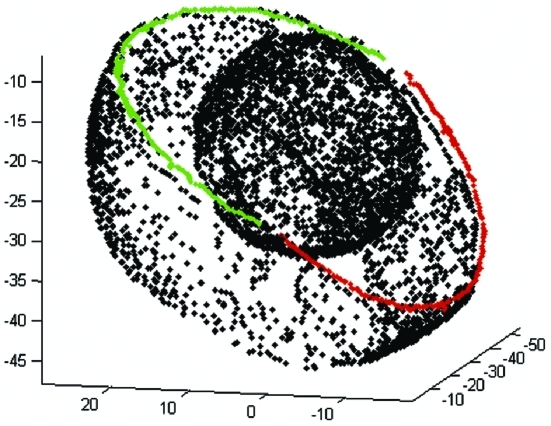

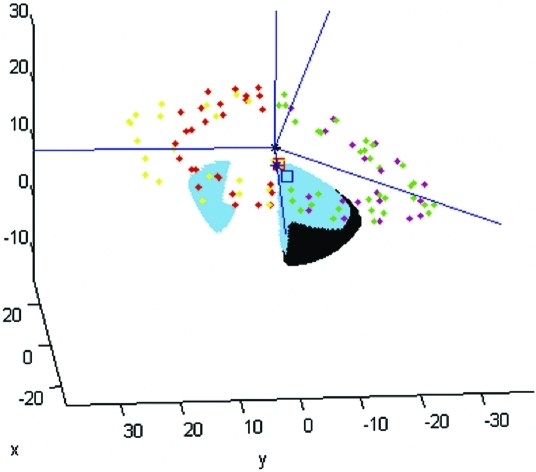
A. Digitized points from CT scan image of head and cup. Values are in mm. B. Example of CMM measurement. Digitized points on cup inner surface and planes, center points before and after wear (square hollow symbols), and coordinate axes (blue lines). Values are in mm.

Image analysis of the CT volumes was performed using a validated 3D volume fusion tool (Maguire et al. 1991, Noz et al. 2001, Gorniak et al. 2003). The first step, for each CT volume, was to designate 3D coordinate points to define (1) the outer surface of the metal shell of the cup, (2) the femoral head surface, and (3) the 2 cup opening faces. Using 3D isosurfaces and a 3D point selection mode, evenly distributed coordinate points were designated that corresponded to the outer surface of the cup (1,000–4,000 points) and the femoral head surface (2,500–5,000 points) (Figure 2A). In order to filter out metal artifacts, all points positioned more than 0.5 mm (i.e. ca. 1 SD) from a spherical surface were eliminated. Using the same strategy, about 200–800 coordinate points on the cup opening faces were also designated and points positioned more than 0.5 mm from a flat surface were eliminated.

The second step was to register the volumes for each pair of CT volumes (pre-wear and post-wear) into a single coordinate system, thus allowing comparison of femoral head and cup positions. The software for calculating wear properties from CT data was based on ideas previously reported (Olivecrona et al. 2003a, b, Jedenmalm et al. 2008), but used new algorithms giving substantially improved numerical accuracy and with several additional utility functions: for instance, algorithms for calculating wear vector angles. The computer program was implemented in Matlab and ComsolScript. Using non-linear optimization methods, planes were fitted to the CT points on the cup opening faces and spheres were fitted to the points on (1) the cup surface, and (2) the femoral head surface. This was done both before and after wear. Local coordinate frames for each were calculated from the normal axes of the plane of the cup opening and the midpoints of the cup surface. Coordinate transformations were then used to transfer all data to a common global coordinate frame, as defined by the head surface sphere, such that the head positions in the worn and unworn cup could be compared. Linear wear was defined as the distance between midpoints of the head sphere in worn and unworn systems. The wear angles (θ and ϕ) were defined as the angles of a standard spherical coordinate system centered in the unworn head.

### Wear testing with measurement by coordinate measurement machine (CMM)

The CMM procedure, just like the CT methodology, consisted of (1) tools for obtaining geometric data, and (2) locally developed software for analyzing the data. However, since it is well established that geometric data obtained by CMM measurements are reliable, the CMM procedure was used as the reference method, together with the gravimetric measurements.

The fundamental principles of CMM have been described previously (Olivecrona et al. 2005). In this particular study, the inside of the polyethylene cups was examined with the CMM method using 8,000 evenly distributed digitizing points; the geometry of the cup opening face planes was digitized using about 30 points (Figure 2B) and that of the femoral head surfaces using 200 points.

The locally developed software for calculating the maximum linear penetration depth, and wear direction, resembled its CT equivalent. The major differences were twofold: (a) instead of calculating the local coordinate axes with the metal part of the cup opening face planes and the cup outer surface, the plastic part of the cup opening face planes and the ultimately unworn (left) side of the inner part of the cup were used; (b) instead of defining the linear wear as movement of the head due to wear, we defined it in terms of the change in the inner, ultimately worn (right) hemisphere of the cup—specifically as the movement of the midpoint of a sphere fitted to that inner hemisphere. The asymmetry in wear was due to the way in which wear is induced by the hip simulator.

### Statistical evaluation of errors

Data were tested for normality using Bland-Altman plots, and the graphical evaluation with line of equality and scatter plots. Confidence intervals were calculated using the Student's t-distribution. Accuracy, repeatability, and bias of the CT method were calculated with the 95% confidence level according to ISO definitions (ISO 3534: 3,11 & 3,14) (ISO 1998). Precision is defined as the closeness of agreement between independent test results obtained under stipulated conditions. For repeatability, as a way to express precision we used the ISO standards, where the method under evaluation is the combined error of acquisition of data with a CT unit and image post-processing (ISO 3534: 3,14) (ISO 1998). Accuracy is defined as the closeness of agreement of a test result (CT in this study) and the accepted reference value (the CMM and gravimetric results). The term accuracy when applied to a set of test results refers to a combination of systematic errors (bias) and random errors (ISO 3534: 3,11) (ISO 1998).

We tested the method for systematic errors by computing an interval estimate of the bias (the mean error and the 95% confidence interval for the mean). Accuracy and repeatability were calculated at the 95% confidence level as described by Ranstam et. al. (2000).

## Results

**Figure 3.**
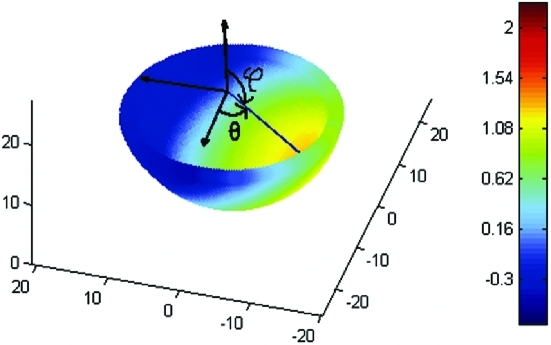
Representative 3D plot with the coordinate axes of linear wear surface distribution and the angles ϕ and θ to the wear vector in blue. [Authors: this sentence is difficult to follow] The color bar represents the magnitude of wear in mm.

An example of the 3D geometry of the CMM measurement and results, with angles ϕ and θ and wear vector, is given in Figure 3.

### Test for systematic differences

**Figure 4.**
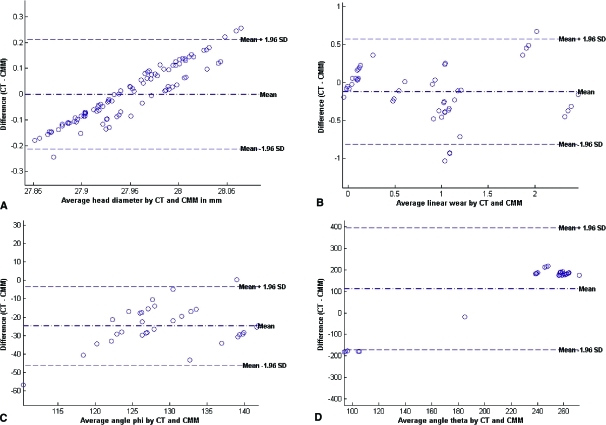
Difference against mean for CT and CMM measurements a) of head diameter b) linear wear c) Angle ϕ d) Angle θ

If the differences obey the normal distribution, then 95% of the differences will lie within the 95% limits of agreement; that is, the mean difference ± 1.96 times the standard deviation. Most of the data points were within these limits. The difference plot for femoral head measurement (Figure 4A) revealed no systematic difference between CT and CMM. The plot for linear wear showed a slight underestimation of the wear (Figure 4B). The plot for angle ϕ (Figure 4C) shows a negative mean difference value that suggests an underestimation with CT, whereas the plot for angle θ (Figure 4D) shows the opposite behavior with a positive mean difference, suggesting an overestimation with CT.

Accuracy and repeatability along with an interval estimate of bias for the experiments are summarized in Table 1. No significant systematic differences were found between repeated CT scans. The landmark points showed a normal distribution.

**Table 1.** Repeatability, accuracy, and interval estimate of bias at 95% confidence level for the proposed CT method

	Repeatability (SD)	Accuracy (SD)	Interval estimate of bias
Linear wear, mm	0.39 (0.19)	0.51 (0.14)	–0.12 ± 0.10
Angle θ, degrees	17 (179)	106 (144)	89 ± 40.9
Angle ϕ, degrees	16 (26)	50 (18)	–33 ± 5.2
Head diameter, mm	0.11 (0.06)	0.20 (0.04)	0.09 ± 0.03

### Analysis of wear

Linear wear of the 12 cups assessed with CMM varied between 0 and 2.54 mm, and the mean difference between CT and CMM measurements of linear wear was –0.12 (95% CI: –0.02 to –0.23) mm (Table 2).

**Table 2.** Mean (SD) linear wear (mm) results from CT and CMM measurements

Cup no.	CT	CMM	Difference
1	0.65 (0.14)	1.55 (0.21)	–0.90
2	0.89 (0.15)	1.23 (0.06)	–0.34
3	0.81 (0.11)	1.15 (0.16)	–0.34
4	0.20 (0.16)	0.08 (0.14)	0.12
5	2.22 (0.12)	2.54 (0.27)	–0.32
6	2.18 (0.13)	1.69 (0.25)	0.49
7	0.01 (0.14)	0.02 (0.23)	–0.00
8	0.17 (0.07)	0.02 (0.24)	0.15
9	1.00 (0.12)	1.26 (0.09)	–0.26
10	–0.01 (0.10)	0.05 (0.08)	–0.06
11	0.46 (0.12)	0.60 (0.08)	–0.14
12	1.04 (0.14)	0.91 (0.13)	0.13
All cups	0.80 (0.13)	0.92 (0.16)	–0.12

Volumetric wear of the 12 cups assessed by weighing varied between –5 and 936 mm^3^. Comparison of the ranking order (lowest to highest wear) showed excellent agreement between gravimetric results and CMM. In the same comparison, CT also agreed well with the gravimetric results except for cups no. 1 and 6.

### Analysis of creep

From CMM measurements, the creep could be estimated from the soak controls (cups no. 4, 7, 8, and 10). The average creep was estimated to be about 0.05 mm.

### Analysis of the head and cup diameters

The head diameters varied between 27.93 and 27.99 mm (SD 0.02 mm) according to CMM. The mean difference in head diameter before wear between CT and CMM measurements was 0.09 (95% CI: 0.06 to 0.12) mm, and after wear it was –0.09 (95% CI: –0.13 to –0.6) mm.

For both head diameter and cup diameter, no systematic measurement differences were found due to different positioning or repeated measurement of the same object. The average difference was 0.001 mm (SD 0.01) and the maximum difference for the same object was 0.15 mm.

The outer diameter of the cup could not be accurately measured with direct measurement since the metal mesh provided an uneven surface, which made automatic measurement with CMM difficult due to the sensitivity of the CMM probe—and it would be too time-consuming to do it manually. However, the outer diameter and the repeatability of the measurements were measured with CT before and after wear (Table 3).

**Table 3.** Measurements (by CT) of outer diameter of cups, and of femoral head diameter

Object	Mean diameter) in mm (SD	Mean SD of repeated measurements
Cup unworn	54.37 (5.14)	0.034
Cup worn	54.77 (5.12)	0.030
Head unworn	28.04 (0.06)	0.041
Head worn	27.86 (0.05)	0.045

The mean diameter was based on all 48 measurements ± the standard deviation. Repeatability was calculated by taking the standard deviation of the 4 measurements of each prostheses and taking the mean of those twelve values. The outer diameter stated by the manufacturer varied between 46 mm and 64 mm, but no bias due to size could be found.

The average difference in outer diameter between worn and unworn cups was 0.40 ± 0.094 mm, whereas for femoral heads the average difference was –0.18 ± 0.068 mm. Since the positions of the cup centers are used as reference points in calculating the wear, it is reasonable to expect a bias of linear wear values of about half the cup diameter, i.e. approximately 0.2 mm.

### Analysis of fitting of the planes

The average standard deviations of points fitted to the planes are tabulated in Table 4. The comparatively large standard deviation of the repeatability was mainly due to one outlier of each plane in cups 1 and 6 (Table 2). If this was removed, the standard deviation of the repeatability was approximately halved.

**Table 4.** Mean standard deviation (SD) of points fitted to the reference planes

Object	Mean fit SD	Mean SD of repeated measurements
Plane 1 unworn	0.195 (0.106)	0.058 (0.075)
Plane 1 worn	0.149 (0.060)	0.038 (0.038)
Plane 2 unworn	0.190 (0.028)	0.020 (0.019)
Plane 2 worn	0.164 (0.053)	0.030 (0.019)

Values in parentheses are SD of mean SD.

## Discussion

We found that it is possible to use CT for wear measurement of acetabular cups. The accuracy was 0.51 mm for linear wear detection. In our previous study on explants (Olivecrona et al. 2005), the accuracy was 0.55 mm. Theoretically, though, we should have clearly improved our method due to the increased number of landmark points and better algorithms. However, compared to the previous study, for estimation of head diameter the accuracy improved from 0.8 mm to 0.2 mm and repeatability improved from 0.3 mm to 0.1 mm, respectively. One explanation for the only small improvement in linear wear estimation is that even though the head diameter estimation was improved, this cup design was more difficult to measure in our image analysis tool due to the metal mesh and the non-spherical outer surface. We also found large deviations in estimation of outer cup diameter, which should be the same before and after exposure to wear. This can be explained by the change in CT acquisition protocol between the “before” and “after” CT scans. This would also be likely to contribute to the lower accuracy and repeatability values. The linear wear data showed 2 outliers: cup no. 1 and cup no. 6. If these were excluded, the accuracy of the proposed method would be improved by 0.01 mm.

In vivo wear rate values for conventional acetabular cups range from 0.1 mm/year to 0.2 mm/year, and total wear at revision is about 1–3.5 mm (Kabo et al. 1993). Acetabular cups with wear rates below 0.1 mm/year can have 90% survivorship after 25 years, whereas none of the cups with wear rates above 0.2 mm/year survive that long (Sochart 1999). This would mean that 3 years after implantation, CT can be used to detect wear in conventional acetabular cups, since anticipated wear would be 0.3–0.6 mm, which CT can indeed dete

Using digitized AP radiographs combined with dedicated computer software, the accuracy was 1.3 mm in a model study (Schewelov et al. 2004), while a study resembling clinical routine has reported errors (of 2 SD) ranging from –1.8 mm to +1.2 mm for 1 mm of wear and from – 4.4 to +0.8 mm for 4 mm of wear after manual assessment of wear by comparing two AP radiographs (Clarke et al. 1976). A drawback of all 2D methods is the risk that the wear vector might be out of the plane of the radiograph, and that the magnitude of the error depends on the degree of anteversion of the cup (Sychterz et al. 1997). The most accurate method today is considered to be radiostereometric analysis (RSA) (Kärrholm et al. 1997). In one study, the accuracy of the RSA digital measurements was 0.42 mm with a mean measurement error of 0.01 mm (Schewelov et al. 2004). However, such methods are not normally available in clinical practice.

Our study was limited by the small number of subjects; therefore, the data should be interpreted with caution. The accuracy of the angles was low due to the difficulty in measurement of the reference plane, since this particular cup design has uneven edges due to the metal mesh. However, in a clinical situation this would not be a problem because the direction of wear could be measured relative to the pelvis.

It is possible to underestimate the wear if the head of the femoral component is not placed into the most worn part of the polyethylene liner during the CT examination. Also, even with modern software, metal artefacts might still complicate interpretation of the image. It is also common that there is a clearance of about 0–0.5 mm between the head and cup diameters, even though it is not stated by the manufacturer (Lewis et al. 2003). This would indicate a possible initial displacement of the head inside the polyethylene liner. In our study, the clearance was measured to be 0.5 mm. Since we measured both before and after wear and calculated the difference, this clearance was taken into account for in this study.

In radiographic techniques including CT, the wear is estimated as the combination of creep and wear. Creep in polyethylene acetabular cups is commonly around 0.1 mm (Isaac et al. 1996), and this is also comparable to our finding with the CMM measurements of the soak controls.

In conclusion, we found that CT has the potential for reliable measurement of linear wear of acetabular cups at a clinically relevant level of accuracy. With appropriate development and automation, this method may help in identifying patients with increased risk of aseptic loosening.

## References

[CIT0001] Clarke IC, Black K, Rennie C (1976). Can wear in total hip arthroplasties be assessed from radiographs?. Clin Orthop.

[CIT0002] Gorniak RJT, Kramer EL, Maguire GQJ (2003). Evaluation of a semi-automatic 3D fusion technique applied to molecular imaging and MRI brain/frame volume data sets. J Med Syst.

[CIT0003] Isaac GH, Dowson D, Wroblewski BM (1996). An investigation into the origins of time-dependent variation in penetration rates with Charnley acetabular cups-wear, creep or degradation. Proc Inst Mech Eng.

[CIT0004] ISO (1998). Accuracy (trueness and precision) of measurement. International standard ISO.

[CIT0005] Jedenmalm A, Noz ME, Olivecrona H (2008). A new approach for assessment of wear in metal-backed acetabular cups using computed tomography: a phantom study with retrievals. Acta Orthop.

[CIT0006] Kabo JM, Gebhard JS, Loren G (1993). In vivo wear of polyethylene acetabular components. J Bone Joint Surg (Br).

[CIT0007] Kärrholm J, Herberts P, Hultmark P (1997). Radiostereometry of Hip Prostheses: Review of Methodology and Clinical Results. Clin Orthop.

[CIT0008] Lewis G, Fencl RM, Caroll M (2003). The relative influence of five variables on the in vitro wear rate of uncrosslinked UHMWPE acetabular liners. Biomaterials.

[CIT0009] Maguire GQJ, Noz ME, Rusinek H (1991). Graphics applied to image registration. IEEE Computer Graphics Appl.

[CIT0010] Mohanty M (1996). Cellular basis for failure of joint prosthesis. Bio Med Mater Eng.

[CIT0011] Noz ME, Maguire GQJ, Zeleznik MP (2001). A versatile functional-anatomic image fusion method for volume data sets. J Med Syst.

[CIT0012] Olivecrona L, Crafoord J, Olivecrona H (2002). Acetabular component migration in total hip arthroplasty using CT and a semi-automated program for volume merging. Acta Radiologica.

[CIT0013] Olivecrona H, Olivecrona L, Weidenhielm L (2003a). Stability of acetabular axis after total hip arthroplasty. Repeatability using CT and a semiautomated program for volume fusion. Acta Radiol.

[CIT0014] Olivecrona H, Weidenhielm L, Olivecrona L (2003b). Spatial component position in total hip arthroplasty. Accuracy and repeatability with a new CT method. Acta Radiol.

[CIT0015] Olivecrona L, Olivecrona H, Weidenhielm L (2003c). Model studies on acetabular component migration in total hip arthroplasty using CT and a semi-automated program for volume merging. Acta Radiologica.

[CIT0016] Olivecrona H, Weidenhielm L, Olivecrona L (2004). A new CT method to measure cup orientation after Total Hip Arthroplasty. A study on 10 patients. Acta Orthop Scand.

[CIT0017] Olivecrona L, Jedenmalm A, Aspelin P (2005). Assessing wear of the Acetabular cup using computed tomography: an ex vivo study. Acta Radiologica.

[CIT0018] Ranstam J, Ryd L, Onsten I (2000). Accurate accuracy assessment. Review of basic principles. Acta Orthop Scand.

[CIT0019] Santavirta S, Anttila A, Aspenberg P (1995). Tekonivelen biokompatibiliteettitutkimus. Finn J Ortop Pharmatol.

[CIT0020] Schewelov T, Sanzén L, Börlin N (2004). Accuracy of radiographic and radiostereometric wear measurement of different hip prostheses: an experimental study. Acta Orthop Scand.

[CIT0021] Sochart DH (1999). Relationship of acetabular wear to osteolysis and loosening in total hip arthroplasty. Clin Orthop.

[CIT0022] Sychterz CJ, Engh CA, Shah N (1997). Radiographic evaluation of penetration by the femoral head into the polyethylene liner over time. J Bone Joint Surg (Am).

[CIT0023] Wang A, Polineni VK, Essner A (1998). Role of proteins and hyaluronic acid in the lubrication and wear of UHMWPE acetabular cups.

[CIT0024] Wang A, Essner A, Schmidig G (2004). The effects of lubricant composition on in vitro wear testing of polymeric acetabular components. J Biomed Mater Res Part B: Appl Biomat.

